# Seroprevalence and factors associated with *Coxiella burnetii* exposure in goats in Moretele

**DOI:** 10.4102/ojvr.v90i1.2071

**Published:** 2023-04-04

**Authors:** Rungano Magadu, Peter N. Thompson

**Affiliations:** 1 Production Animal Studies, Faculty of Veterinary Science, University of Pretoria, Pretoria, South Africa

**Keywords:** seroprevalence, *Coxiella burnetii*, goats, ELISA, risk factors, zoonosis

## Abstract

Infection with *Coxiella burnetii* causes significant economic impact and poses zoonotic risk to people exposed to livestock, yet few studies in South Africa have assessed seroprevalence of *C. burnetii* infection and no information is available for goats. Very little information is available regarding risk factors and outcomes of *C. burnetii* infection in peri-urban farming areas where widespread mixing of ruminants occurs. This study estimated the seroprevalence of *C. burnetii* infection among communally farmed goats in an area adjacent to the densely populated Gauteng province. Sera were collected from 216 goats in 39 herds, and questionnaires were completed to establish management practices as potential risk factors. *C. burnetii* antibody testing was done by ELISA. Thirty two out of 216 goats tested positive for *C. burnetii* antibodies and the overall seroprevalence, adjusted for sampling weights and clustering, was 18.4% (95% confidence interval [CI]: 12.2% – 23.5%). The intraclass correlation coefficient was 0.06, indicating low-to-moderate clustering. Multiple logistic regression showed age was significantly associated with seropositivity, with higher seroprevalence in animals ≥ 19 months old (26%) than animals ≤ 6 months old (6%) (odds ratio [OR]: 6.6; *p* = 0.010). We concluded *C. burnetii* infection is common in goats in Moretele and a potential cause of abortion in goats and poses the potential zoonotic disease risk.

**Contribution:**

Despite the threats posed on animal health and productivity, scant information is published on *C. burnetii* in South Africa. This research established preliminary estimates of *C. burnetii* seroprevalence. The research is original from a South African perspective, relevant to Africa and focused on infectious disease in livestock.

## Introduction

Q fever is a zoonotic disease caused by the small gram-negative coccobacillus, *Coxiella burnetii*, an obligate intracellular bacteria belonging to the order Legionellales and bacterial family Coxiellaceae (Arricau-Bouvery & Rodolakis 2005; Menadi et al. 2020). *C. burnetii* infection is endemic worldwide, except in New Zealand, and affects a wide range of domestic and wild mammals, fish and some arthropods (Huang et al. 2021; Karagul, Malal & Akar 2019; Kazar 2005). Domestic ruminants such as cattle, goats and sheep are considered the reservoirs of human infection and infected animals shed *C. burnetii* in the faeces, urine, milk and vaginal discharge (Guatteo et al. 2011; Menadi et al. 2020). More than 40 species of ticks have been shown to be capable of multiplying *C. burnetii* in their GIT mucosal cells and then passing out viable bacteria in their faeces (Knap et al. 2019; Körner et al. 2020). Ticks are thought to play an important role in the maintenance of infection within domestic and wildlife hosts and infection rates of up to 10% have been reported in some studies (Bellabidi et al. 2020; Körner et al. 2020). Animal vaccines against *C. burnetii* are licensed for use in some European countries but are not registered or used in South Africa and most of Africa (Bauer et al. 2021; Rahaman et al. 2019).

*C. burnetii* infections of animals are mostly subclinical but when clinical disease occurs, it is characterised by abortions, particularly in sheep and goats, sometimes on an epidemic scale, and by stillbirths, premature birth and birth of weak offspring (Madariaga et al. 2003; Woldehiwet 2004). In infected ewes and nanny goats, *C. burnetii* infection can cause infertility and metritis, while infected cows can develop mastitis due to the pathogen (Menadi et al. 2020). Affected sheep shed the organism transiently, and they experience higher rates of abortions than cattle (Waag 2007). In humans, about 60% of *C. burnetii* infections are associated with asymptomatic disease or mild, self-limiting, flu-like symptoms with occasional complications such as endocarditis, meningitis and atypical pneumonia (Njeru et al. 2016).

*C. burnetii* infection in animals is of particular importance in developing countries due to poor tick control and mixing of ruminant herds in communal pastures (Vanderburg et al. 2014), which amplify the risk for animals becoming infected. It has been shown that people who are positive for human immunodeficiency virus (HIV) are at higher risk of developing severe signs and symptoms of Q fever (Patil & Regunath 2021) and as South Africa has the highest population of people living with HIV in the world, with 7.7 million HIV-positive people (Parker et al. 2020) out of a population of about 58 million (De Micco et al. 2022), the potential impact of Q fever in humans can be high. The consumption of unpasteurised dairy products, which is widespread in South African communal areas, is one of the transmission modes of *C. burnetii* infection from animals to humans (Gale et al. 2015). Despite the effects of *C. burnetii* infection on productivity in ruminants and the associated human health risk, very little has been done to assess infection rates in animal and human populations, especially in South African peri-urban communal farming areas. A study of *C. burnetii* seroprevalence in cattle in Gauteng province (formerly Transvaal province) estimated *C. burnetii* antibody prevalence at 8% (Gummow, Poerstamper & Herr 1987) and Adesiyun et al. (2020) reported a seroprevalence of 38% in cattle in a rural area of South Africa at a wildlife-livestock interface. However, there are no reports on *C. burnetii* seroprevalence in South Africa for goats, which are very commonly kept in communal and peri-urban areas throughout South Africa due to their hardy nature. In South Africa, there is a lack of active surveillance programmes and a lack of epidemiological data, and *C. burnetii* infection in animals is not listed as a controlled or notifiable disease according to the *Animal Diseases Act of South Africa (Act 35 of 1984)* (Mangena et al. 2021).

Elsewhere in Africa, *C. burnetii* infection in animals and humans has been documented in countries such as Ghana, Cote d’Ivoire, Togo and Burkina Faso but, as in South Africa, there are very few active surveillance programmes and hence epidemiological data on infections in humans and animals are sparse (Johnson et al. 2019). As it is a neglected zoonotic disease with potentially serious effects on human health and animal economics, *C. burnetii* surveys are necessary to establish the impact on human and animal populations, particularly in communal areas.

The objective of this study was to estimate the prevalence of exposure to *C. burnetii* among the goat population in the villages of the Moretele communal farming area, a peri-urban area adjacent to the densely populated province of Gauteng, South Africa. In addition, associated risk factors for seropositivity and potential outcomes of exposure to *C. burnetii* were also assessed.

## Materials and methods

### Study area

**FIGURE 1:**
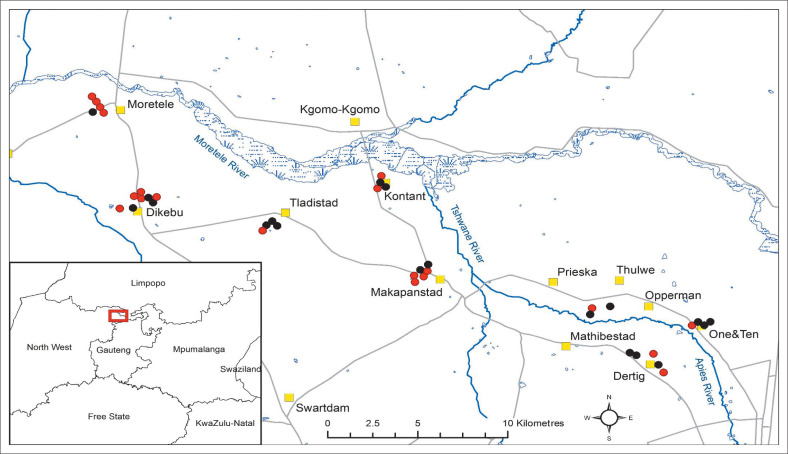
Map of study area on the boundary between Gauteng and the North West province, South Africa, showing the sampling locations of goats tested for *C. burnetii* antibodies. Grey lines indicate roads; yellow squares indicate villages; red dots indicate properties on which *C. burnetii*-positive goats were found and black dots are *C. burnetii*-negative properties.

The study area was located between latitudes 25.142°S to 25.285°S and longitudes 27.970°E to 28.253°E. Eight villages in the North West province, near the boundary of Limpopo and Gauteng provinces of South Africa, were selected for sampling (Figure 1). The villages are located in the floodplain catchment areas of the Moretele, Apies and Tshwane Rivers. The terrain in the study area is mainly flat, semi-arid and the predominant vegetation types are sour bushveld, savannah and springbokvlakte thornveld (Dynamic Intergrated Geo-Environmental Services 2012). The average annual rainfall in the study area is 565 mm, with rain falling in the summer months between October and March, and the annual average temperature is 25 °C (Accuweather 2022; Dynamic Intergrated Geo-Environmental Services 2012). The human population density in Moretele is about 818/km^2^ (StatsSA 2022). The study area is peri-urban, located about 30 km north-west of Hammanskraal township and 20 km north of Soshanguve, which is the largest township in Pretoria (Tshwane). Most livestock owners in the area have small herds of cattle and/or goats and make use of communal pastures and drinking points, resulting in frequent mixing of animals from different herds. There is generally minimal veterinary intervention, mostly comprising vaccinations by state veterinary services personnel.

### Study design and sampling

The study was conducted in December 2018 and was designed to estimate the seroprevalence of *C. burnetii* in goats using multi-stage random sampling. Information on goat population distribution by household was obtained from state-employed animal health technicians responsible for animal health in the study area. Only villages where several goat owners owned five goats or more were selected. Within selected villages, random sampling of households by a hat draw method was followed by random sampling of goats within households after obtaining written informed consent from each farmer.

### Sample size calculation

Sample size was calculated based on the equation for a sample size to estimate prevalence with 95% confidence in an infinite population assuming simple random sampling (Thrusfield 2007):

$$n=\frac{1.96^{2}P_{exp}(1-P_{exp})}{d^{2}}$$ [Eqn 1]

where *n* is the required sample size, *P*_*exp*_ is the expected prevalence and *d* is the desired absolute precision. A minimum of 110 goats were calculated for sampling based on an expected prevalence of 20% and a desired precision of 7.5%. The design effect (DEFF) for clustering was calculated as follows:

DEFF=1+ρ(m−1), [Eqn 2]

where *m* is the cluster size and *ρ* is the intracluster correlation coefficient (ICC) (Bennett et al. 1991), set at 0.2 as it is unlikely to exceed this value for most infectious diseases (Otte & Gumm 1997). For an average cluster size (*m*) of five goats sampled per household, DEFF was calculated as 1.8 and multiplied by the sample size for simple random sampling to give an adjusted minimum required sample size of 198 goats, to be sampled from 40 households.

### Blood sample collection

Ten millilitre of blood from each goat was collected by jugular venepuncture with 20-gauge needle into a vacutainer serum tube. Samples were transported on ice in a cooler box to the laboratory where they were centrifuged at 1500 g for 10 min at room temperature to separate. The serum samples were stored at -20 °C until serological testing.

### Collection of data

A questionnaire was used to collect data from goat owners. The questionnaire collected information in two parts with the first part being individual goat-specific information such as age, sex, breed, history of kidding, history of abortion and the origin of each goat sampled. Flock-specific information made up the second part of the questionnaire and obtained information such as flock abortions in the previous 12 months, deaths in the previous 12 months and management practices such as dipping of goats, routine buying and selling of goats and the use of injectable tetracyclines in the goat flocks.

### Laboratory testing

Testing for *C. burnetii* antibodies was done using the LSIVet^TM^ Ruminant Q fever – serum/milk enzyme-linked immunosorbent assay (ELISA) (Priocheck, Leylstad, the Netherlands), which is an indirect ELISA kit for the detection of phase 1 and phase 2 anti-*C. burnetii* antibodies in ruminant serum or milk. The kit was used according to the manufacturer’s recommendations. Validations performed by INRA and Life Technologies report a diagnostic sensitivity (Se) of 87% and diagnostic specificity (Sp) of 100% for this test (De Oliveira et al. 2018). For each sample, the sample/positive (S/P) ratio was calculated as:

$$\text{S}/\text{P}=[{\text{OD}}_{\text{Sample}}-{\text{OD}}_{\text{NC}}]/[{\text{OD}}_{\text{PC}}-{\text{OD}}_{\text{NC}}],$$ [Eqn 3]

where OD_Sample_ was the optical density of each sample tested, OD_NC_ was the average optical density of the negative control and OD_PC_ was the average optical density of the positive control. The *C. burnetii* antibody titre was then calculated as Titre = S/P × 100. The results were interpreted as follows: ≤ 40 was negative, 40–100 was mild positive, 100–200 was moderate positive and > 200 was strong positive.

### Statistical analysis

For the analysis, mild, moderate and strong positives were all regarded as positive results. Age was categorised into terciles (≤ 6 months, 7–19 months, > 19 months). Prevalence of *C. burnetii* antibodies and 95% confidence intervals was calculated and adjusted for sampling weights and clustering using the *svy* (survey) commands in Stata 14 (StataCorp, College Station, Texas, United States). Prevalences and confidence intervals were then also adjusted for the reported diagnostic Se and Sp of the ELISA using the formula (Rogan & Gladen 1978):

TP=(AP+Sp−1)/(Se+Sp−1), [Eqn 4]

where TP is true prevalence and AP is apparent prevalence. The ICC (*ρ*) was calculated as:

$$\rho =\sum_{i=1}^{K}{\left\{Y_{i}+(Y_{i+}-1)-2P(n_{i}-1)Y_{i+}+n_{i}(n_{i}-1)P\right\}}^{2}/\sum_{i=1}^{K}\left\{n_{i}(n_{i}-1)P(P-1)\right\},$$ [Eqn 5]

where *K* is the number of herds, *Y*_*i+*_ is the number of seropositive animals in herd *i, n*_*i*_ is the number of animals tested in herd *i* and *P* is the overall unadjusted seroprevalence.

Univariate analysis of potential risk factors for seropositivity was done by cross-tabulation and the Fisher’s exact test. Factors associated with a positive test outcome at *p* < 0.2 were selected for inclusion in a multivariable logistic regression model, which was then developed by backward elimination until variables remaining in the model were significant at *p* < 0.05. Herd size was included as a random effect and adjustment for sampling weights and clustering in the multistage survey design was done using the *svy* command in Stata^®^. Univariate associations between seropositivity and its potential consequences, such as abortions, were assessed using logistic regression and odds ratios were calculated. Statistical significance was assessed at *p* < 0.05.

### Ethical considerations

The research protocol was approved by the University of Pretoria Animal Ethics committee (Approval number V001-18), the University of Pretoria Faculty of Humanities Research Ethics committee (Approval number GW20170928HS) and the Department of Agriculture, Forestry and Fisheries (Approval numbers 12/11/1/1/6 and 12/5/1).

## Results

A total of 216 goats belonging to 39 goat owners (average of 5.5 goats per herd) were sampled across the eight villages. The total number of *C. burnetii* antibody-positive goats was 32/216, with estimated animal-level prevalence, after adjustment for sampling weights, clustering and diagnostic Se and Sp, of 18.4% (95% CI: 12.2% – 23.5%). Seropositive goats were found in 20/39 household herds, giving an estimated herd prevalence of 51% (95% CI: 35% – 68%). The intraclass correlation co-efficient (ICC) was 0.06, indicating low to moderate clustering of *C. burnetii* seropositivity within the tested goat herds.

Univariate associations (*p* < 0.2) of potential predictor variables with *C. burnetii* seropositivity were found for age category (*p* = 0.001) and breed (*p* = 0.159) (Table 1). These variables were therefore selected for inclusion in the multivariable model.

**TABLE 1:** Factors associated with seropositivity to *Coxiella burnetiii* amongst communally-farmed goats in Moretele District: Univariate analysis.

Variable and level	*n*	% positive	*p*
**Herd size**			0.764
< 15	70	17	
15–19	79	13	
> 19	67	15	
**Age class**			0.001
0–6 months	67	6	
7–19 months	64	9	
> 19 months	85	26	
**Sex**			0.286
Female	156	17	
Male	60	10	
**Breed**			0.159
Angora	1	100	
Boergoat	46	11	
Kalahari Red	18	22	
Mixed	143	15	
Saanen	8	0	
**Origin**			0.233
Born on premises	202	16	
External origin	14	0	
**Buy animals**			0.966
No	114	15	
Yes	102	15	
**Dipping**			0.840
No	64	14	
Yes	152	15	

**Total**	**216**	**15**	**-**

For the multivariable analysis, due to small category sizes, breed was re-categorised as Boergoat, Mixed breed and Other (included Kalahari red, Angora and Saanen). In the final model (Table 2), the odds of seropositivity increased with age, being 6.6 times greater in goats > 19 months than in those ≤ 6 months (95% CI: 1.6–26.7; *p* = 0.010). Prevalence also varied by breed, with the lowest odds of seropositivity in Boergoats and the highest in ‘Other’ (*p* = 0.033).

**TABLE 2:** Mixed-effects logistic regression model of factors associated with *Coxiella burnetii* seropositivity.

Variable and level	Odds ratio	95% CI (OR)	*p*
**Age class**			
0–6 months	1 (base)	-	-
7–19 months	1.7	0.3–8.5	0.497
> 19 months	6.6	1.6–26.7	0.010
**Breed**			
Boergoat	1 (base)	-	-
Mixed	2.6	0.6–11.6	0.203
Other	6.3	1.2–33.6	0.033

CI, confidence interval; OR, odds ratio.

Univariate analysis of potential consequences of infection (Table 3) showed that goats with a history of abortion were significantly more likely to be *C. burnetii*-seropositive (OR: 4.6; 95% CI: 1.1–20.2; *p* = 0.042). In addition, goats in herds that had experienced more than two abortions in the past 12 months tended to have greater odds of seropositivity than goats in herds that had experienced no abortions (OR: 2.9; 95% CI: 0.9–6.8; *p* = 0.071).

**TABLE 3:** Potential consequences of *Coxiella burnetii* infection in communally-farmed goats in Moretele District and their association with seropositivity to *C. burnetii*.

Variable and level	*n*	% positive	Odds ratio (OR)	95% CI (OR)	*p*
**Goat with history of kidding**					
No	26	15	1.0		
Yes	83	22	1.5	0.5–5.0	0.487
**Goat with history of abortion**					
No	101	18	1.0		
Yes	8	50	4.6	1.1–20.2	0.042
**Abortions in herd past 12 months**					
0	139	12	1.0		
1–2	50	16	1.4	0.5–3.4	0.501
> 2	27	26	2.5	0.9–6.8	0.071
**Retained foetal membranes in herd**					
No	210	14	1.0		
Yes	6	33	3.0	0.5–17.1	0.216
**Tetracyclines used in herd past 12 months**					
No	145	16	1.0		
Yes	71	13	0.8	0.3–1.8	0.537
**Animals sold from herd**					
No	33	18	1.0		
Yes	183	14	0.8	0.3–2.0	0.555
**Mortalities in herd past 12 months**					
No	94	20	1.0		
Yes	122	11	0.5	0.3–1.2	0.119
**Slaughter and consumption of meat**					
No	54	9	1.0		
Yes	162	17	2.0	0.7–5.4	0.191

## Discussion

This study is the first to document seroprevalence of *C. burnetii* in goats in South Africa, showing widespread exposure to the pathogen in the study area, with estimated animal-level and herd-level seroprevalences of 18.4% and 51%, respectively. This finding is of public health significance, since it indicates frequent exposure to an important zoonotic pathogen amongst livestock in close proximity to densely-populated urban areas.

Muleme et al. (2017) reported an animal seroprevalence of 25% – 43% for *C. burnetii* within herds of dairy goats in Victoria, Australia in a study conducted during an outbreak between 2012 and 2014. This higher seroprevalence is likely due to more intensive farming systems in dairy goats, with higher population density and concentrated kidding seasons, which are the highest risk periods for uninfected animals becoming infected by infected placentas and uterine fluids (Muleme et al. 2017). A study in sheep and goats in northern Egypt showed an overall animal seroprevalence of *C. burnetii* of 15% – 20%, similar to the current study, and the authors suggest that the seroprevalence of *C. burnetii* is generally higher in goats than in sheep (Selim et al. 2018). The similarity in seroprevalence could be due to similarities in management practices and climatic conditions, with low rainfall, hot temperatures and possibly abundance of ticks being common to the two study areas.

Moretele communal area, as with other rural communities, has most goats on communal grazing and this results in widespread mixing of goat herds, spread of infection between herds and a high herd seroprevalence, compared to private farms with no herd mixing. However, the herd seroprevalence of *C. burnetii* in this study (51%) was lower than the 80% reported from northern Jordan (Lafi et al. 2020). The higher farm level seroprevalence in Jordan could be explained by the arid nature of pastures in Jordan where the nomadic goats have to walk long distances to find food and also, the more frequent mixing of goat herds in these sparse pastures leads to greater horizontal transmission. In contrast, herd-level seroprevalence was only 8.0% for intensively-farmed dairy goat herds in a study in Victoria, Australia (Tan 2018), where there was likely absence of mixing of goat herds and more frequent use of acaricides. A study in communal areas in Senegal, where w*C. burnetii* deoxyribonucleic acid (DNA) was assayed in ticks, showed infection rates of up to 37.6% of the sampled ticks (Mediannikov et al. 2010). We postulate that high tick infection rates could have contributed to the higher herd seroprevalence seen in this study, compared to those by Tan (2018). Another study in Algeria, though in cattle, by Menadi et al. (2020) reported a herd seroprevalence of 45.6%, similar to the 51% found in this study. Although this was in cattle, this is possibly due to similarities in animals mixing in communal pastures, similar tick control practices and sharing communal water sources as well as closely related climatic conditions of Algeria and semi-arid Moretele.

There was evidence that increasing age was significantly associated with increased odds of seropositivity and animals > 19 months of age were 6.6 times more likely to test positive for *C. burnetii* antibodies than were animals < 6 months of age. This age association was also reported in an Egyptian study where animals > 4 years of age had higher seroprevalence than younger ones (Klemmer et al. 2018) as well as in another study in Kurdistan province of Iran where animals 3 years and older were more likely to test positive (Fakour, Jamali & Ahmadi 2021). It is to be expected that, as with most infectious diseases, the seroprevalence will increase with age due to cumulative exposure to *C. burnetii* over time.

In this study, there was no significant association of *C. burnetii* seropositivity with herd size. This contrasts with the findings of Rizzo et al. (2016) in north-west Italy, where the seroprevalence in sheep and goat herds larger than 12 goats was about three times higher than in smaller herds. Rizzo et al. (2016) postulate this could be due to crowding which amplifies the risk of horizontal transmission of *C. burnetii*. In our study, larger herd size did not necessarily mean that there was more crowding at pasture, due to the absence of fencing in the extensive grazing system on communal pastures.

The ICC of 0.06 demonstrates low to moderate clustering of *C. burnetii* within the tested goat herds. *Coxie lla burnetii* is a contagious pathogen, therefore, the presence of one infected goat likely means several other goats in the same herd are also infected, and clustering is to be expected. Communal grazing, over and above the risk of aerosol transmission, pasture contamination with faecal material and urine, and vaginal discharge contaminated with *C. burnetii*, also carries the risk of infected ticks moving within and between goat herds (Angelakis & Raoult 2010). Because in communal systems transmission occurs easily between herds, clustering of contagious diseases within herds is likely to be less pronounced than where herds are kept separate, resulting in only low to moderate clustering, consistent with our finding. In contrast to our study, Adesiyun et al. (2020) reported a much higher degree of clustering of *C. burnetii* seropositivity in communally farmed cattle at dip tanks adjacent to the Kruger National Park (ICC = 0.57). The difference can be ascribed to the fact that, in that study, clustering was assessed at the dip tank level, not at the level of herds within dip tanks, and animals from different dip tanks are very unlikely to mix, compared with animals from different herds within communal areas.

This study showed an association between seropositivity for *C. burnetii* and the occurrence of abortion, at both the individual and the herd level. This confirms the importance of *C. burnetii* as an abortigenic agent that could have significant economic impact in livestock herds. It should be considered as a possible differential diagnosis when investigating single or outbreaks of abortions in ruminant animals in South Africa, and precautions should be taken to prevent zoonotic transmission when handling abortion products, as for other important zoonotic abortigenic agents that occur in South Africa, such as brucellosis and Rift Valley fever.

## Conclusion

*C. burnetii* infection is widespread in the Moretele municipality of South Africa and is present in more than 50% of goat herds. It is likely that the seroprevalence is similar in many other communal areas throughout the country, due to similarities in management practices, as well as the widespread transport of goats between different parts of the country. Further studies in South Africa in cattle, sheep and goats, and in people that are occupationally at risk should be done to determine the true extent of infection with *C. burnetii* in animals and the occupational risk of infection for humans. Studies into the prevalence of *C. burnetii* in cases of abortion, mastitis and metritis should be designed to assess the impact of disease on various production systems for cattle, sheep and goats. Once comprehensive data on disease prevalence and impacts in South African livestock farming is elucidated, optimal management practices to mitigate against the spread of *C. burnetii* withinin intensive and extensive animal husbandry systems can be suggested. These management practices may include effective tick control, purchasing animals from herds that regularly test and minimising mixing of herds, although some of these are not always easily applicable in communal areas. Awareness campaigns should be conducted to educate farmers on the risks of *C. burnetii*, appropriate protective measures to prevent human exposure, and the benefits of controlling this infection in animals.
